# Compliance of Disease Awareness Campaigns in Printed Dutch Media with National and International Regulatory Guidelines

**DOI:** 10.1371/journal.pone.0106599

**Published:** 2014-09-08

**Authors:** Teresa Leonardo Alves, Auramarina F. Martins de Freitas, Martine E. C. van Eijk, Aukje K. Mantel-Teeuwisse

**Affiliations:** 1 World Health Organization Collaborating Centre for Pharmaceutical Policy and Regulation, Utrecht Institute for Pharmaceutical Sciences (UIPS), Utrecht University, Utrecht, The Netherlands; 2 Independent Consultant, Utrecht, The Netherlands; University of Mainz, Germany

## Abstract

**Background:**

The European legislation prohibits prescription-only medicines' advertising but allows pharmaceutical companies to provide information to the public on health and diseases, provided there is no direct or indirect reference to a pharmaceutical product. Various forms of promotion have become increasingly common in Europe including “disease-oriented” campaigns.

**Objectives:**

To explore examples of disease awareness campaigns by pharmaceutical companies in the Netherlands, by assessing their compliance with the World Health Organization (WHO) Ethical Criteria for medicinal drug promotion and the Dutch guidelines for provision of information by pharmaceutical companies.

**Methods:**

Materials referring to health/disease and treatments published in the most widely circulated newspapers and magazines were collected from March to May 2012. An evaluation tool was developed based on relevant underlying principles from the WHO ethical criteria and Dutch self-regulation guidelines. Collected disease awareness advertisements were used to pilot the evaluation tool and to explore the consistency of information provided with the WHO and Dutch criteria.

**Findings:**

Eighty materials met our inclusion criteria; 71 were published in newspapers and 9 in magazines. The large majority were news items but 21 were disease awareness advertisements, of which 5 were duplicates. Fifteen out of the 16 disease awareness campaigns were non-compliant with current guidelines mainly due to lack of balance (n = 12), absence of listed author and/or sponsor (n = 8), use of misleading or incomplete information (n = 5) and use of promotional information (n = 5). None mentioned a pharmaceutical product directly.

**Conclusion:**

Disease Awareness Campaigns are present in Dutch printed media. Although no brand names were mentioned, the lack of compliance of disease awareness campaigns with the current regulations is alarming. There were information deficiencies and evidence of information bias. A key concern is that the context in which the information is provided, mostly through indirect referral, is likely to support treatment with the sponsor's product.

## Introduction

In 1988, the World Health Organization established the Ethical Criteria for Medicinal Drug Promotion, defining promotion as “all informational and persuasive activities of manufacturers and distributors that affect the prescription, supply, purchase and/or use of medicinal drugs” [1]. While not legally binding, these criteria include a set of guiding principles that can be adapted to national circumstances.

Advertising of prescription drugs to the public – also known as direct-to-consumer advertising (DTCA) – is controversial and only allowed in the United States and New Zealand. European legislation prohibits advertising of products that have prescription-only status, aiming to protect public health. Despite this prohibition, however, manufacturers are using an increasing array of techniques to advertise these medicines to the public both directly and indirectly [2].

Media and communication channels are key influencers of consumer decisions, helping to shape consumers' information and options, also on health and treatment [3]. Media can also exert a powerful influence over human behaviours and public policy [4]. Health topics are often covered in printed media and they can include factual information on diseases and conditions but also treatment information of promotional nature [5], [6].

In Europe, pharmaceutical companies are explicitly allowed to provide general information to the public on human health and diseases, as long as there is no reference, even indirectly, to a specific medicinal product [7]. This provision enables companies to run unbranded ‘disease-awareness’ or ‘help-seeking’ advertisements [8]. These materials draw viewers' attention to certain health conditions by focusing on symptoms and suggesting the public to ‘ask their doctors’ for newly available treatment information [9]. Such campaigns are not subject to any specific regulations governing pharmaceutical promotion, nor pre-clearance. They represent a grey area in regulation since regulators are reluctant to consider them to be product-specific promotion unless they include explicit links to branded information [10].

Disease awareness campaigns (DACs) can educate the public about disease, make consumers aware of untreated health problems and lead them to seek effective care at earlier stage, thus leading to better health [11]. Advocates consider disease awareness campaigns to be particularly important for under-diagnosed diseases [12]. However, concerns have been raised about the quality and nature of the information being provided to the public in disease awareness campaigns [13]. Proponents of direct to consumer advertising claim that it empowers consumers by stimulating discussions with physicians, enabling patients to obtain needed treatment at an earlier stage and improving adherence. Evidence shows, nevertheless, that exposure to advertisements increases prescribing volume and patient demand and that it shifts prescribing into less cost-effective choices. In addition, there is no evidence of improved adherence, nor treatment quality or early provision of needed care [14]. In a similar trend, albeit scarce, there is evidence that disease awareness campaigns can lead to increases in consultation rates and prescriptions for the advertisers' product [15]. If campaigns support use of newer, more expensive products with least well understood benefit-harm profiles, over cheaper, well-known, older medicines, they can lead to increases in consultations, inappropriate prescribing and more adverse drug reactions and drug-induced harm, as well as increases in hospitalisations, thus affecting both quality and costs of care [9]. While much research has been done in other areas of traditional drug promotion, far less is known about how these campaigns influence both physicians and the public, or on their compliance with the current regulatory framework [16].

In the Netherlands, a self-regulatory approach is used to oversee medicines' advertising [17]. In April 2011, the Foundation for the Medicinal Products' Advertising Code (CGR) published a set of guidelines on the provision of disease and treatment information about prescription-only medicines by pharmaceutical companies to the public, thus aiming to define the boundary between information and advertising [18].

Nevertheless, there is no instrument available to assess the compliance of disease awareness campaigns with the provisions included in the WHO Ethical Criteria for Medicinal Drug Promotion or in the Dutch self-regulation guidelines.

Evaluating the quality and nature of the information provided in disease awareness campaigns is very relevant to policy discussions at European level. The proposal for a European directive on information to the general public on medicinal products subject to medical prescription, presented by the European Commission in December 2008, foresaw changes to the regulations on advertising. It contemplated an expanded role for the pharmaceutical industry in the provision of information on prescription medicines directly to the public [19]. This study aims to inform future European policies regulating the dissemination of disease and treatment information to the public by the pharmaceutical industry. This article assesses the frequency of occurrence of disease awareness campaigns in printed media in the Netherlands and measures their compliance with current guidelines.

## Objectives

The aim of our research was threefold:

1. To assess the frequency of occurrence of medicines' promotion and disease awareness campaigns in printed media in the Netherlands.

2. To develop a user-friendly instrument to assess the compliance of disease information campaigns.

3. To use this instrument to measure compliance of disease information campaigns, including those disseminated by pharmaceutical companies, in Dutch printed media.

## Methods

### 1. Assessing disease-awareness campaign frequency in major print media

We examined high-circulation print media, which included three paid daily newspapers, three free daily newspapers and eight paid monthly magazines [20]. Data collection took place over three months (March to May 2012 inclusive). The three free newspapers were collected at train stations whereas the 14 paid publications were accessed in public libraries. Two authors (AMF and TLA) independently selected materials based on the inclusion criteria outlined below. If there were disagreements, these were to be resolved by consensus. Consensus was reached in all cases.

Our **inclusion criteria** were based on an interpretation of legal provisions, which prohibit direct and/or indirect reference to a pharmaceutical product. Firstly, we included all materials which addressed health and treatment issues. Materials on nutraceuticals, homeopathic products, over-the-counter medicines and vaccines were excluded, as they are governed by different legislation. Secondly, we selected all materials which covered one or more of the following four sets of linked information: (1) symptoms/health issues (for prevention purposes)/diseases/conditions AND a specific prescription-only medicine or a therapeutic drug class; (2) symptoms/health issues (for prevention purposes)/diseases/conditions AND a doctor or website referral/a description of a drug's mechanism of action or a suggestion to seek further treatment; (3) name or the logo of a pharmaceutical company AND mention of a symptom/problem/condition or referred to a website; and (4) reference to disease management programmes and discussion of adding another medical product to the ongoing treatment regimen. Finally, materials were then separated into two groups: disease awareness campaigns (Group I) – which included no author and were to be assessed using the instrument – and news items (Group II) – editorial content which included an author or was attributable to the news desk.

Additional descriptive data which were also recorded included: type of publication where materials were found (Paid or Free); printed media frequency; topic; reference to non-pharmaceutical interventions; reference to changes in the quality of life (either positive or negative); referral to visit a physician; reference to a clinical expert; referral to a website; reference to a patient organization or support group; reference to a brand-name, use of company's name or logo, and reference to the availability of a new medicine or treatment option.

### 2. Instrument development

The instrument (originally developed in Dutch and then translated into English) is based on seven relevant criteria from the WHO Ethical Criteria for Medicinal Drug promotion [1] and the KOAG/CGR guidelines [17]. These were identified by overlapping the relevant provisions within the two sets of regulatory guidelines that can be used to judge the content and quality of disease-oriented information (Table 1). These are promotional information, misleading or incomplete information, use of fear, inadequate language, lack of balance, use of testimonials and absence of listed author and/or sponsor. These criteria were translated into evaluation statements to judge whether or not a principle is being adequately applied (compliant, non-compliant and not applicable). An option to insert additional comments was also included. A reference to a company's name or logo in a disease awareness campaign was not considered sufficient for a material to be deemed non-compliant.

**Table 1:** Overlap between relevant provisions within the WHO Ethical Criteria for Medicinal Drug Promotion and the CGR Guidelines for provision of information on prescription medicines.

WHO Ethical Criteria	CGR Guidelines	Relevant criteria identified
Article 6. Definition of promotion: “all informational and persuasive activities by manufacturers and distributors, the effect of which is to induce the prescription, supply, purchase and/or use of medicinal drugs.”	Introduction. Definition of promotion: “all informational and persuasive activities by manufacturers and distributors, the effect of which is to induce the prescription, supply, purchase and/or use of medicinal drugs.”	Promotional information
Article 7. “Promotional material should not be designed so as to disguise its real nature.”	Introduction. “Instances whereby prescription medication or pills are being mentioned without indicating the drug's brand name or company name” are considered indirect reference, for example when naming the active ingredients or the drug's mechanism of action.	
Article 9. “Scientific and educational activities should not be deliberately used for promotional purposes.”	Article 5. “Information may not encourage irrational use of prescription medicines nor the search for unnecessary treatment.”	
Article 14b. “Advertisements to the public should not generally be permitted for prescription drugs or to promote drugs for certain serious conditions that can be treated only by qualified health practitioners.”	Article 6. “Information may not directly or indirectly lead to the choice of a particular medicine from different available treatments.”	
Article 7. “Advertisements may claim that a drug can cure, prevent, or relieve an ailment only if this can be substantiated. … All promotion-making claims concerning medicinal drugs should be reliable, accurate, truthful, informative, balanced, up-to-date, capable of substantiation and in good taste. They should not contain misleading or unverifiable statements or omissions likely to induce medically unjustifiable drug use or to give rise to undue risks.”	Article 3. “Information may not be misleading. The information provided must comply with the most recent evidence and practice standards. The information must be factually correct and may not contain any misleading elements.”	Misleading or incomplete information
	Article 17. “No comparison is allowed between relevant treatments and medicines that suggests that the effects of a treatment with a prescription drug are better or equal than those of another relevant treatment or drug.”	
	21.2 b) “No single option for treatment is to be highlighted, for instance by using words, colours or images, different font types, markings or any other elements. ”	
	Article 21.2 d) “Treatments should be cathegorised based on acceptable formats. For instance using therapeutic classes or categories, or through therapeutic guidelines. Using expressions such as “most recent, or new is better, most commonly used, is not allowed.”	
	Article 23. “Information should be displayed objectively and neutrally and must not contain information which relates directly to a specific treatment. When reference is made to specific treatment guidelines, the source must be listed…References to scientific literature should also be published…”	
Article 14: “While they [advertisements] should take account of people's legitimate desire for information regarding their health, they should not take undue advantage of people's concern for their health.”	Article 9.“Information should not aim nor encourage the public to seek unnecessary treatment, advice or further examination; nor on the other hand refrain the public from seeking treatment, advice or further examination.”	Use of Fear
	Article 5. “Information may not encourage irrational use of prescription medicines nor the search for unnecessary treatment.”	
Article 15. “Language which brings about fear or distress should not be used.”	Article 4. “Information should not boost or amplify feelings of fear and superstition and should be displayed realistically.”	
	Article 20. “The information may not be unjustified, unnecessarily alarming or misleading images of changes to the human body resulting from illness or disease.”	
Article 29. “The wording …if prepared specifically for patients, should be in lay language on condition that the medical and scientific content is properly reflected.”	Article 7. “Information should be tailored to the average consumer and have understandable language. Medical and scientific terms should be avoided as much as possible, to avoid confusion.”	Inadequate Language
Article 7. “All promotion-making claims concerning medicinal drugs should be reliable, accurate, truthful, informative, balanced, up-to-date, capable of substantiation and in good taste. They should not contain misleading or unverifiable statements or omissions likely to induce medically unjustifiable drug use or to give rise to undue risks… Comparison of products should be factual, fair and capable of substantiation”.	Article 9.“Information should not aim nor encourage the public to seek unnecessary treatment, advice or further examination; nor on the other hand refrain the public from seeking treatment, advice or further examination.”	Lack of Balance
	Article 17. “No comparison is allowed between relevant treatments and medicines that suggests that the effects of a treatment with a prescription drug are better or equal than those of another relevant treatment or drug.”	
	Article 21. “Information should be as balanced and complete as possible. It should reflect the state-of-the-art. When providing information, all relevant factors should be taken into account. All information should be equally displayed both in content and layout, with the same amount of detail.”	
	Article 21.2 c) “The positive and negative effects of a treatment are not to be emphasized in such a way that the pros or the cons of a given treatment are highlighted”.	
	Article 21.2 d) Information about different therapeutic interventions can be provided. In that case, all relevant treatments should be named, including pharmacotherapy and other interventions, such as adjustments to lifestyle, nutrition and habits. Relevant treatments are the standard of care provided, as per treatment guidelines. Completeness ensures that no information is deliberately omitted. When enumerating all the pharmacotherapeutic options for treatment, all the relevant prescription drugs for the specific treatment are to be mentioned.”	
Article 7. “Promotional material should not be designed so as to disguise its real nature.”	Article 18. “Testimonials should portray the opinion or experience of the user truthfully (not that of a professional or any other public figure). They should not include any comparison of the user's situation before and after drug treatment…Before/after testimonials are not allowed because they can lead the public into false expectations regarding the speed of the treatment's effects”.	Testimonials
Article 9. “Scientific and educational activities should not be deliberately used for promotional purposes.”		
Article 7. “Advertisements may claim that a drug can cure, prevent, or relieve an ailment only if this can be substantiated. … All promotion-making claims concerning medicinal drugs should be reliable, accurate, truthful, informative, balanced, up-to-date, capable of substantiation and in good taste. They should not contain misleading or unverifiable statements or omissions likely to induce medically unjustifiable drug use or to give rise to undue risks.”	Article 22. “Each message is to contain the name of the person responsible for the information”.	Absence of Source/Author
	Article 23. “Information may refer to scientific studies and results…The source must always be included. The studies and the results that are mentioned must always come from other sources than the medicine's producer and should be verifiable…” “Information should be displayed objectively and neutrally and must not contain information which relates directly to a specific treatment. When reference is made to specific treatment guidelines, the source must be listed…References to scientific literature should also be published…”	

Three external reviewers (pharmaceutical policy researchers) tested the instrument using three examples of disease awareness campaigns by the pharmaceutical industry, previously published in European printed media. As a result, five statements were altered. Changes included merging, division or rewording of statements. The final instrument is included as Table S1.

### 3. Assessing compliance of disease awareness campaigns with guidelines

As the instrument is based on legal guidelines, the existence of a single non-compliant statement is sufficient to consider the material to be non-compliant. Two authors (TLA and AMF) independently assessed each of the seven criteria for all disease awareness campaigns and differences in scoring were discussed and resolved by consensus. Any remaining disagreements were then adjudicated to a third author (AMT). The frequencies of the information provided in both groups were measured using the risk ratio (RR). When information was absent, a cell in Table 2 obtained a zero and no RR could be calculated. This was dealt by adding 0.5 to every cell in Table 2 to be able to calculate an estimate of the RR [21]. Whenever possible, data analysis was conducted using SPSS version 20.0 (SPSS Inc. Chicago, Illinois, USA).

**Table 2:** Material Characteristics.

Type of publication	Group 1 Disease awareness campaigns (n = 21) (% within group)	Group 2 News items (n = 59) (% within group)	Risk Ratio (95% CI)
***All publications***			
Paid	5 (24%)	43 (73%)	
Free	16 (76%)	16 (27%)	**2.8 (1.7; 4.5)**
***Publication frequency***			
Daily	5 (24%)	38 (64%)	
Weekly	2 (9%)	3 (5%)	
Monthly	0 (0%)	4 (7%)	
Occasionally	14 (67%)	14 (24%)	
***Health Supplements***	15 (71%)	13 (22%)	**3.4 (1.9; 5.6)**

*Information included*	Group 1 Disease awareness campaigns (n = 21) (% within group)	Group 2 News items (n = 59) (% within group)	Risk Ratio (95% CI)
Non-pharmaceutical interventions in addition to therapy	5 (31%)	19 (32.2%)	0.9 (0.4; 2.2)
Suggestion to visit the general practitioner	6 (37%)	10 (17%)	2.21 (0.9; 5.1)
Reference to a Clinical expert	3 (19%)	14 (24%)	0.8 (0,3; 2.4)
Website	13 (81%)	9 (15%)	**5.3 (2.8; 10.1)**
Patient or support group	6 (37%)	9 (15%)	2.45 (1.0; 5.9)
Pharmaceutical company	6 (37%)	8 (8%)	**2.8 (1.1; 6.8)**
Brand name	0 (0%)	10 (17%)	**0.3** * **(0.0; 2.2)**
New medicine or treatment option	2 (12%)	15 (25%)	0.5 (0.1; 1.9)

* RR calculated by adding 0.5 to all cells.

## Results

### 1. Assessing disease-awareness campaign frequency

On average six materials covering disease and treatment information were published per week. A total of 80 materials were collected, 59 of which were news items (73,8%), whereas 21 were disease awareness campaigns (26.3%) (Table 2). Five of these disease awareness campaigns were duplicates - published in different printed media - leaving 75 materials for further description and 16 materials for the compliance analysis.

Overall (n = 80) the seven most commonly mentioned conditions were: allergies and respiratory diseases (n = 22; 28%), diabetes (n = 7; 9%), cardiovascular diseases (n = 5; 6%), cancer (n = 5; 6%), contraception (n = 4; 5%), Attention Deficit Hyperactivity Disorder (ADHD) (n = 4; 5%) and pain (n = 4; 5%). Within disease awareness campaigns, allergies and respiratory diseases, and contraception were common topics. Most notably, all the disease awareness duplicates regarded allergies and respiratory diseases. One disease awareness campaign was sponsored by a patient organisation.

Disease awareness campaigns were significantly more frequent in free publications (RR  = 2.8, 95% CI 1.7; 4.5) and in health-related supplements (RR  = 3.4, 95% CI 1.9; 5.6). When comparing news to disease awareness campaigns as to the information provided, the latter were more likely to mention a pharmaceutical company (RR  = 2.8, 95% CI 1.1; 6.8), a website (RR  = 5.3, 95% CI 2.8; 10.1) or a visit to the general practitioner (RR  = 2.2, 95% CI 0.9; 5.16) but less likely to include a brand-name (RR  = 0.3, 95% CI 0.04; 2.2) (Table 2).

### 2. Assessing compliance of DACs with guidelines

**Figure 1:**
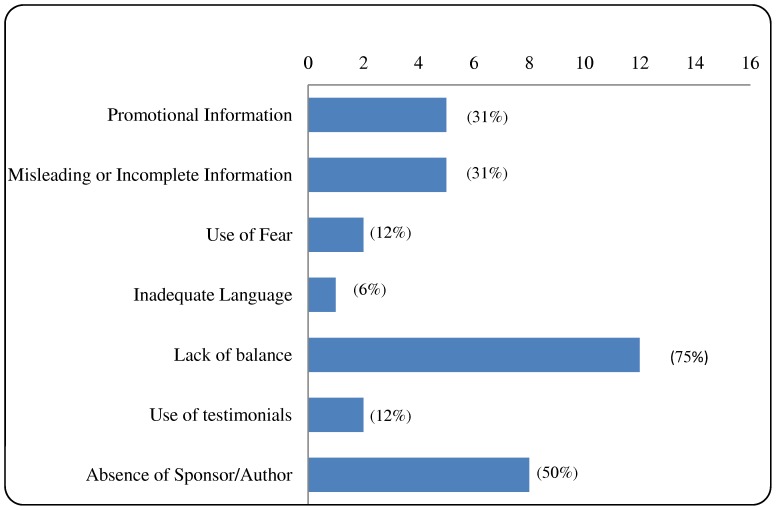
Non-compliance of disease awareness campaigns (n = 16) per key criteria. Fifteen out of the sixteen materials assessed were non-compliant with the guidelines. Non-compliance was more frequent due to lack of balance, absence of listed author and/or sponsor, use of promotional information and use of misleading or incomplete information.

The initial inter-rater agreement in the assessment of overall compliance was of 88%; disparities between assessors were arbitrated by the fourth author. Fifteen out of the sixteen materials assessed were non-compliant with the guidelines. Non-compliance was more frequent due to lack of balance, absence of listed author and/or sponsor, use of promotional information or use of misleading or incomplete information (Table 3, Figure 1). Interestingly, most instances of non-compliance with the misleading or incomplete information criterion involved a lack of references.

**Table 3:** Examples of non-compliance per key criteria from the disease awareness campaigns.

KEY criteria	Problem identified	Example *(CONDITION)*
**Promotional information**	Reference to pharmaceutical products to treat a condition or disease in combination with: the name, logo and website of a pharmaceutical company; or a website for a disease awareness campaign; or quick response codes to dedicated websites.	“We are an international company with expertise in lung diseases…we develop innovative pharmaceutical solutions…” *(Respiratory diseases)*
		A dedicated website is mentioned in big and bold typeface, as well as the name, logo and website of a pharmaceutical company. (*Contraception)*
		“X strives respond to challenging medical conditions through innovative approaches. Pain treatment is one of our priorities… physical and emotional challenges borne by pain patients are key. Our R&D programme seeks alternative solutions to fight pain.” *(Pain)*
		“Our website helps you to choose the best treatment for your lifestyle. You will be able to find information about all therapies…and also do a test to help you select a suitable treatment.” *(Kidney Failure)*
		“Do you have, or someone close to you has, urinary incontinence? Have a look at our different links: (company) website; your digital logbook for your mobile phone; your online digital logbook to share with your doctor.” *(Urinary incontinence)*
**Misleading or incomplete information**	No reference is provided on the sources of information provided about prevalence of disease.	“One out of every 8 adults in the Netherlands has high cholesterol”. (*Cardiovascular diseases)*
		“One out of each 10 Dutch has asthma …“one out of every 5 Dutch has hay fever”. *(Allergies)*
		“More than 5000 people get post-traumatic dystrophy every year.” *(Post-traumatic dystrophy)*
		“Approximately 40.000 Dutch suffer from renal disease. There are several treatments available for this debilitating disease.*” (Kidney Failure)*
		“One third of those who suffer from migraine in the Netherlands do not get the appropriate treatment…2.5 million Dutch suffer from migraine.” (*Migraine)*
**Use of fear**	Reference to disability caused by the disease, either through text or picture.	An image of a disabled hand is used. *(Post-traumatic dystrophy)*
		“Besides the pain…migraine also has implications for society…it costs the Netherlands 1.7 billion per year… I have seen people who cannot fulfil their dreams…. That is terrible… *(Migraine)*
**Inadequate language**	Uses medical terminology	“Perinasal inflammation…abscesses…metabolic diseases…” *(Alarm signals)*
**Lack of balance**	More emphasis on the benefits of pharmaceutical treatment than risks. Symptoms are accentuated by layout and/or enumeration. Risk factors are portrayed as diseases. Treatment is accentuated.	“This disease can have a great impact on the individual and its environment. It disturbs your daily life. It is important to diagnose it at an earlier stage, so that treatment can begin quickly. Therapy includes anti-inflammatory drugs and painkillers.” *(Post-traumatic dystrophy)*
		“These symptoms can seem mild, but they can have a great impact on your daily life, at school or at work, and even disturb your sleep patterns”.
		“Symptoms such as shortness of breath, cough and wheeziness result in an asthma attack…Red and itchy eyes, running nose, stuffy nose, sneezes and tiredness can have serious implications.” *Allergies)*
		Symptoms are referred to in headings in big and bold typeface. *(Allergies) (Alarm signals) (Urinary incontinence)*
		“…when you have high cholesterol, you have a higher risk of developing cardiovascular diseases… you can reduce that risk by…treating your high cholesterol levels. Have a look at our new website about healthy living with lower cholesterol”. (*Cardiovascular diseases)*
		“Now women are able to choose a pill that contains a natural hormone and a progestogen. This natural hormone is easily absorbed by the body…this pill has a neutral effect on acne, weight-gain and blood pressure…your periods will be shorter and lighter…” *(Contraception)*
		Contraception is mentioned on six occasions in big and bold typeface. *(Contraception)*
		The sentence: “I (do not) want a pill” and the address of a dedicated website are included in big and bold typefaces. *(Contraception)*
		“…suffering from migraine, days in a row, a pain impossible to bear…with nausea, and sensitivity to light and noises…seek a good treatment…Medicines play an important role…we advise patients to try two different triptans…” (*Migraine)*
**Use of testimonials**	Specialist mentions treatment and specific drug classes	“The doctor can prescribe anti-histamines…or corticosteroids… immunotherapy can be considered an option”. (*Allergies)*
	A comparison is made of the patient's experience before and after treatment with a specific drug.	“I had tummy and back aches with another pill. I visited my doctor and together we have chosen a new pill with a different ingredient. That has helped”. “ The first pill I took caused weight-gain and emotional changes. My GP then prescribed a lighter pill and I am feeling fine”. *(Contraception)*
**Absence of author and/or sponsor**	No author and/or sponsor identified.	*(Allergies), (Alarm signals), (Cardiovascular diseases), (Contraception), (Migraine)*

Most notably, five out of the sixteen materials included the logo or name of a pharmaceutical company, referred to a particular condition and mentioned a treatment indirectly. Other four materials discussed a condition and indirectly a treatment, while including a referral to a website sponsored by a pharmaceutical company. Table 3 provides examples of non-compliance aggregated per key criteria and disease awareness campaign topic.

## Discussion

In this study we have shown that there is a focus on disease and treatment information in printed media in the Netherlands, both through news items and disease awareness campaigns. The majority of disease awareness campaigns identified during our study period did not comply with the WHO ethical criteria nor with the current Dutch self-regulation guidelines.

Most collected materials on health and treatment were news items (74%). On average there were at least five news items published every week and seven disease-awareness campaigns published every four weeks. Our results seem to indicate that pharmaceutical companies often opt to reach a wider audience by publishing their unbranded product advertisements in free media outlets, most notably in dedicated health-supplements. The frequency of occurrence of disease awareness campaigns observed in our study is consistent with the results of an Australian study, where a total of sixty campaigns were identified in popular women's magazines over eleven months [22]. From these, fifteen contained a corporate brand or logo – a result also similar to ours.

The findings on low compliance are worrying, since serious information deficiencies in disease awareness campaigns result in information bias. A key concern is that the context in which the information is provided will be biased towards supporting treatment with the sponsor's product. One third of the disease awareness campaigns in our study referred readers to their physicians. Disease awareness campaigns can stimulate patients' intentions to make requests to doctors for prescription medicines products, increase consultation rates as well as prescriptions for the advertiser's product [15], [23]. A survey in Australia has shown that 26,9% of the 800 patients enquired had approached their GP to discuss a treatment they had heard about in the media. Half of the patients reported that their inquiry had resulted in a treatment; more than forty-eight percent of those receiving treatment, reported being prescribed a medicine [5]. This has serious implications for general practitioners and regulators.

There is evidence that self-regulation of drug promotion is ineffective. A recent Swedish study demonstrated an overall system failure compounded by lax oversight, regulation lags, and low fines for violations [24]. The authors concluded that the current regulatory regimes have failed to deter industry from providing unreliable information. Researchers have raised concerns in the United States about the effects of indirect medical advertising, claiming that medical decisions based on such influences, as manipulated by advertisers, are likely to result in worse outcomes for patients, and have called for indirect advertising to be curtailed [25].

In contrast, researchers in Australia have concluded that the value of disease awareness campaigns could be improved if regulations and guidelines stipulated disease information requirements [23]. Our research suggests that in the Netherlands – where such guidelines do exist – pharmaceutical companies are aware of the regulatory grey area that disease awareness campaigns represent – and of their subsequent limited regulatory response – thus circumventing the law and exploring new avenues in unbranded product advertising.

The indirect reference to a treatment in association with the name or the logo of a pharmaceutical company – observed in five disease awareness campaigns – constitutes unbranded product advertisement and seems to be in contravention of European law [7], [17]. Our results are consistent with those of a 2009 study in the Netherlands which analysed 41 websites offering health information in the Dutch language: 32 were either hosted or sponsored by a pharmaceutical company, and 23 (72%) contravened national regulations by referring directly or indirectly to a specific prescription medicine [26].

The absence of an identifiable advertiser or sponsor was one of the main factors of non-compliance in our sample. This might be deliberate, as pharmaceutical companies face a real threat of litigation from unsubstantiated marketing claims. Their goal is to raise awareness about a condition and the availability of a treatment, but to leave the responsibility for a decision to the patient, who should “talk to the doctor the advantages and disadvantages of this new therapy” [27]. There is evidence that the rate of diagnoses of specific conditions increases during associated advertising campaigns [14]. A randomized controlled trial using standardized patients found that if patients requested an advertised brand, they were as likely to receive a prescription whether they had the condition that the product treated, depression, or milder life problems not requiring a medicine (‘adjustment disorder’) [28].

More than half of the disease awareness campaigns in our study (62%) referred to websites, some of which seemed independent at first glance, but were sponsored and/or maintained by one or more pharmaceutical companies. Bearing in mind the growing interest in online health information and that consumers are more likely to seek out more prescription drug information after exposure to advertising, as well as to engage in more communication with doctors about prescription drugs, the evaluation of the content and quality of disease awareness websites should also be envisaged [29].

Disease awareness campaigns have been identified as a form of disease mongering or “widening the boundaries of treatable illness in order to expand markets for those who sell and deliver treatments.” [30], [31] A recent commentary by Schwartz and Woloshin provides a template for how disease awareness campaigns work, using three basic strategies: lowering the bar for diagnosis (turning ordinary life experiences into conditions that require medical diagnoses), raising the stakes so that people want to get tested, and spinning the evidence about drug benefits and harms [16].

While seasonality might have influenced the conditions being mentioned in the materials – namely allergies and respiratory diseases - it is unlikely that it would have affected their quality. Contraception was one of the key topics covered in disease awareness campaigns. A new contraceptive pill was launched into the EU market in May 2012 [32]. This might indicate a potential marketing strategy of the marketing authorisation holder to draw attention to their new product. Newspaper readers were also amply exposed to information on diabetes. This might have been related with the inclusion of linagliptine into the Dutch reimbursement list [33].

One of the main limitations of our study has been the small sample of unique advertisements, due to the monitoring and inclusion process. A longer data collection window of a full calendar year would have allowed better sampling and extended statistical analysis.

The dynamics of disease awareness campaigns are intricate and deserve closer scrutiny by physicians, consumers and regulators [27], [34]. While our proposed instrument has not been systematically evaluated, it represents an attempt to translate the relevant provisions included in the WHO Ethical Criteria for Medicinal Drug Promotion and the Dutch self-regulation guidelines into measurable operational components [1], [17], [18]. Further validation and testing are needed, to verify our tool's consistency and reliability.

## Conclusions

We have demonstrated that disease awareness campaigns are present in Dutch printed media. Their compliance with current self-regulation guidelines is low, which warrants the need for further research into the effects of these campaigns. The use of our instrument could help identify disease awareness campaigns of promotional nature and further encourage effective monitoring and implementation of the regulation by competent authorities.

## Supporting Information

Table S1 **Instrument to assess the compliance of disease and treatment information disseminated by pharmaceutical companies to the public with the WHO Ethical Criteria on Pharmaceutical Promotion and the Dutch ‘KOAG/CGR guidelines for information on prescription medicines.**
(DOCX)
