# Supplementary material for: Compliance of Disease Awareness Campaigns in Printed Dutch Media with National and International Regulatory Guidelines
Source: PLoS One. 2014 Sep 8;9(9):e106599. doi: 10.1371/journal.pone.0106599 (PMC4157805; doi:10.1371/journal.pone.0106599)
Supplement: Table S1 — Instrument to assess the compliance of disease and treatment information disseminated by pharmaceutical companies to the public with the WHO Ethical Criteria on Pharmaceutical Promotion and the Dutch ‘KOAG/CGR guidelines for information on prescription medicines. (DOCX) [file pone.0106599.s001.docx]

| **TABLE S1 - INSTRUMENT to assess the compliance of disease and treatment information disseminated by pharmaceutical companies to the public with the WHO Ethical Criteria on Pharmaceutical Promotion and the Dutch ‘KOAG/CGR guidelines for information on prescription medicines** | | | | | | |
| --- | --- | --- | --- | --- | --- | --- |
| **Criteria** | **Compliant (C)** | **Non-compliant (NC)** | **C** | **NC** | **Not applicable** | **Comments** |
| 1.**Promotional information** | NO (in)direct reference to a pharmaceutical intervention, by for example: Naming a therapeutic class *or* Naming or displaying a specific medicine *or* Using a picture and/or link suggesting intervention (ex. “stop-now.com”) *or* Naming a treatment in general for which only one drug is available | (In)direct reference to a pharmaceutical intervention, by for example: Naming a therapeutic class *or*  Displaying a specific medicine *or* Using a picture and/or link suggesting intervention (ex. “stop-now.com”) or Naming a treatment in general for which only one drug is available |  |  |  |  |
|  | *AND/ OR* | *IN COMBINATION WITH:* |  |  |  |  |
|  | NO reference to a pharmaceutical company is made | A reference to a pharmaceutical company *OR* Naming a drug by brand name (e.g. company’s logo or name mentioned in the text) |  |  |  |  |
| **2. Misleading or incomplete information** | The information about pharmaceutical treatment meets national clinical guidelines | The treatment presented is off label *and / or* does not meet national clinical guidelines |  |  |  |  |
|  | *OR* | *OR* |  |  |  |  |
|  | Both a new therapy AND old therapy (in line with national clinical guidelines) are mentioned. (If the new therapy is the only one mentioned and no suggestion is made about its superiority, select N.A) | A comparison is made between several pharmaceutical treatments, highlighting the superiority of a given treatment, which does NOT meet clinical guidelines. (Additional information: a treatment is portrayed in a positive light and adjectives such as ‘new’, ‘spectacular’ and ‘effective’ are used) |  |  |  |  |
|  | Claims or statements made about Prevalence *or* Incidence *or* Costs *or* Disease gravity *or* Disease burden are accompanied by reference(s) to available evidence (e.g. current guidelines and peer-reviewed journals) | NO reference is provided on the sources of the information provided about: Prevalence *or* Incidence *or* Costs *or* Disease gravity *or* Disease burden |  |  |  |  |
| **3. Use of Fear** | There is NO reference to fatal events or disability caused by not treating the disease (through a pharmaceutical intervention) | The text *and/ or* a picture refers fatal events or disability resulting from the non-treatment on the disease |  |  |  |  |
| **4. Inadequate Language** | Medical and scientific terminology are correctly described and interpreted. | Use of medical and scientific terminology without providing a (correct) explanation or interpretation |  |  |  |  |
| **5. Lack of Balance** | Treatment benefits and harms are accurately and proportionally portrayed. (Additional information: Beneﬁts referred can include symptom control or elimination, prevention of recurrence, or eliminating disease. Harms/Risks can include side effects, complications and adverse drug reactions) | More emphasis on the benefits of pharmaceutical treatment than on its risks. (Additional information: Beneﬁts referred can include symptom control or elimination, prevention of recurrence, or eliminating disease. Harms/Risks can include side effects, complications and adverse drug reactions) |  |  |  |  |
|  | Sufficient and correct information is provided to clearly distinguish between a condition requiring drug treatment and normal health and/or milder conditions not requiring drug treatment | Non-pharmaceutical interventions are erroneously omitted |  |  |  |  |
|  |  | *OR* |  |  |  |  |
|  |  | Risk factors are portrayed as disease(s) |  |  |  |  |
|  |  | *OR* |  |  |  |  |
|  |  | Natural ageing processes such as osteoporosis (at 50 +), menopause, arteriosclerosis etc. are portrayed as disease(s) |  |  |  |  |
|  | Symptoms *and/or* treatment are not emphasized | Symptoms  *and/ or* treatment are accentuated by layout *and/ or* enumeration |  |  |  |  |
| **6. Use of Testimonials** | There is no professional, scientist or public figure supporting the treatment with a specific drug | The opinion or experience of a professional, scientist or a public figure is given in support of treatment with a specific drug |  |  |  |  |
|  | *AND/ OR* | *AND/ OR* |  |  |  |  |
|  | NO before / after treatment comparison involving an individual patient | A comparison is made of the patient’s experience before and after treatment with a specific drug |  |  |  |  |
| **7. Absence of Author/sponsor** | The author and/or sponsor is /are clearly stated. | The author and/or sponsor is/are not mentioned. |  |  |  |  |

| **The following questions are considered additional information and therefore do not affect the material’s assessment.** | | | | |
| --- | --- | --- | --- | --- |
| **Descriptive characteristics:** | | **Yes** | **No** | **Comments** |
| *Website* | Is there a reference to a website? |  |  |  |
| *Treatment* | Is the public being referred to visit their GP/ medical specialist/ pharmacist for additional information, a diagnosis and/or possible treatment? |  |  |  |
| *Self-diagnosis* | Is there any reference to a symptom check-lists or questionnaire? |  |  |  |
